# A Novel GNAS Mutation in a Patient with Ia Pseudohypoparathyroidism (iPPSD2) Phenotype

**DOI:** 10.3390/genes14020324

**Published:** 2023-01-26

**Authors:** Anna Gorbacheva, Tatyana Pogoda, Viktor Bogdanov, Victoriya Zakharova, Rustam Salimkhanov, Anna Eremkina, Galina Melnichenko, Natalia Mokrysheva

**Affiliations:** 1 Department of Pathology of the Parathyroid Glands and Mineral Metabolism Disorders, Endocrinology Research Centre, 117292 Moscow, Russia; 2 Laboratory of Medical and Molecular Genetics, Endocrinology Research Centre, 117292 Moscow, Russia; 3 Endocrinology Research Centre, 117292 Moscow, Russia

**Keywords:** GNAS protein, pseudohypoparathyroidism, genetic diseases, case report

## Abstract

Pseudohypoparathyroidism (PHP) is a heterogeneous orphan disease characterized by multihormonal resistance and several phenotypic features. In some cases, PHP is caused by a mutation in the *GNAS* that encodes the alpha subunit of the G protein, one of the key transmitters of intracellular signals. A correlation between the genotype and phenotype of patients with *GNAS* mutations has not yet been described. This often makes diagnosis, drug prescription, and timely diagnosis difficult. Information about GNAS functioning and the impact of specific mutations on the clinical course of the disease is limited. Establishing of the pathogenicity by newly identified *GNAS* mutations will expand the understanding of this gene functioning in the cAMP signaling pathway and may become the basis for personalized treatment. This paper provides a clinical description of a patient with the Ia PHP phenotype caused by a previously unknown mutation in *GNAS* (NC_000020.11(NM_000516.7)): c.719-29_719-13delinsACCAAAGAGAGCAAAGCCAAG in the heterozygous state. Verification of the pathogenicity of the detected mutation is also described.

## 1. Introduction

*GNAS* is a complex locus located on the long arm of chromosome 20. Several products can be transcribed from this locus, among them alpha subunit of the G protein (GNAS protein), the 55 kDa chromogranin-like neuroendocrine specific protein (NESP55), the extra-large isoform of the stimulatory G alpha subunit of the XLαs protein, as well as the non-coding antisense transcript; *GNAS* expression can also lead to A/B transcript methylation [1]. Gαs initiates a cascade of intracellular reactions that transmit a signal from the ligand to the cell nucleus. This is one of the most important post-receptor signaling pathways [2]. All *GNAS* genes, including Gαs, are expressed in a monoallelic way (i.e., from the maternal or paternal allele) [3,4]. This is possible due to imprinting through differentially methylated *GNAS* regions. NESP55 and Gαs are expressed from the maternal allele, while XLαs, antisense transcript AS, and transcript A/B are expressed from the paternal allele. Expression of Gαs in most tissues can happen from both alleles, while in some of them (proximal renal tubules, thyroid gland, gonads, pituitary somatotrophs, and paraventricular and dorsomedial nuclei of the hypothalamus) it is expressed only from the maternal allele [5,6,7,8].

Due to the monoallelic expression of Gαs in these tissues, they are affected by the inactivating mutations on the maternal allele, resulting in the pseudohypoparathyroidism (PHP) phenotype.

There are several types of PHP classified in accordance to certain mutations, inheritance, and clinical course [9]. The type which stems from the inactivating mutations of the *GNAS* gene, classified as PHP type IA (according to the latest classification, iPPSD2), is characterized by multihormonal resistance and the Albright’s hereditary osteodystrophy (AHO) phenotype including obesity, brachydactyly, shortening of the IV/V metacarpal and metatarsal bones, rounded face, short neck, subcutaneous calcifications, short stature, mental retardation, etc. To date, more than 180 different mutations in the *GNAS* that lead to PHP Ia are known. At the same time, two-thirds of these mutations are unique, i.e., were found only once [10].

There is no strict correlation between the genotype and phenotype in *GNAS* mutations. Patients with similar mutations may have different phenotypes of the disease [11]. The functioning of *GNAS* and the consequences of specific mutations remain poorly understood. In this regard, the study of the PHP pathogenesis in the case of previously unknown mutations is required to expand our understanding of *GNAS* functioning. The description of patients with PHP and their relatives (so-called “nuclear” families) with previously unexplored mutations will both determine the personalized treatment approach for each patient and expand world experience in this hereditary disease.

In this work, we present a clinical case of a patient with typical PHP Ia phenotype (iPPSD2), caused by a previously unknown mutation in the *GNAS*, and explore the molecular mechanism leading to the protein defect.

## 2. Materials and Methods

Editorial Policies and Ethical Considerations: This work was approved by Local Ethics Committee.

Peripheral blood samples were obtained from the proband, his parents, and his sister. DNA was extracted from leukocytes by standard methods and exons 1–13, and the flanking intron sequences of the human GNAS gene were amplified by PCR using PCR-Komplect (Synthol, Moscow, Russian Federation) following the manufacturer’s protocol. PCR products were sequenced using a Big-Dye Terminator v3.1 Cycle Sequencing kit (Thermo Fisher Scientific, Waltham, MA, USA) and an ABI PRISM 3500 Genetic Analyzer (Applied Biosystems, Thermo Fisher Scientific, Waltham, MA, USA).

In order to visualize the observed mutation, amplicons corresponding to 10 and 11 exons with adjacent intronic regions of the GNAS gene were separated by electrophoresis in 6% polyacrylic gel for 1 h and 15 min with constant 180 V.

Total RNA was extracted from peripheral blood samples using an RNAEasy Kit (Qiagen, Hilden, Germany), according to the manufacturer’s protocol. cDNA was obtained by reverse-transcription polymerase chain reaction using an ImProm-IITM Reverse Transcription System (Promega, Madison, WI, USA) and oligo(dT)21 primer, according to the manufacturer’s protocol. The cDNA was normalized by GAPDH and used as matrix for amplification of the region corresponding to 8–13 exons of the GNAS mRNA using Phire-DNA Polymerase(Thermo Fisher Scientific, Waltham, MA, USA). Amplicons were separated by electrophoresis in 6% polyacrylic gel. In case we observed the only band, amplicon was directly subjected to Sanger sequencing. In other cases, the bands were cut, and the material extracted from gel and subjected to Sanger sequencing.

Sequences of all primers are available in Supplementary Table S1.

## 3. Results

### 3.1. Case Description

Patient T., a 19-year-old male, admitted to the Department of Parathyroid gland pathology in the Endocrinology Research Centre (Moscow, Russian Federation) with general weakness, fatigue during physical exercise, and excessive weight.

### 3.2. Anamnesis Morbi

The medical history was collected mainly from the patient’s mother and the medical records because of the patient’s mental retardation.

Patient T. is a child from the third physiological pregnancy. His elder brother died at the age of 4 years for unspecified reasons. During his life, he was diagnosed with PHP (the diagnosis was performed without genetic testing). The patient’s elder sister is clinically healthy and has two healthy daughters. The patient’s mother had symptoms of hyposomy (small height—155 cm, miniature hands and feet), but without clinical manifestations of PHP.

Hypocalcemia, hyperphosphatemia, increased serum concentrations of PTH, TSH, and psychomotor retardation were detected in T. at the age of 8 months. At the age of 2 years (in 2003), according to the clinical data, PHP was diagnosed, but genetic testing was not performed. From 2003 to 2008, the patient received alfacalcidol 1.2 mcg daily without normocalcemia achievement: Ca total was 1.9 mmol/L (2.15–2.55). Alfacalcidol was replaced with cholecalciferol 25,000 IU daily. Data on further therapy and laboratory results are presented in Table 1.

At the same time, levothyroxine was prescribed with a gradual dose titration from 12.5 mcg/day in 2003 to 150 mcg/day in 2010. In addition, at the age of 4 years the patient was diagnosed with diabetes insipidus, and desmopressin was administered.

According to brain MSCT in January 2016, multiple areas of mineralization were visualized in the cortex and in the basal nuclei of both hemispheres. Other information considering complications of PHP or comorbidities was not available.

At admission, the patient received desmopressin 120 mcg daily sublingually in the evening (data on the duration of this dosage were absent), cholecalciferol 25,000 IU twice a day, and levothyroxine 150 mcg daily.

### 3.3. Physical Examination

**Figure 1:**
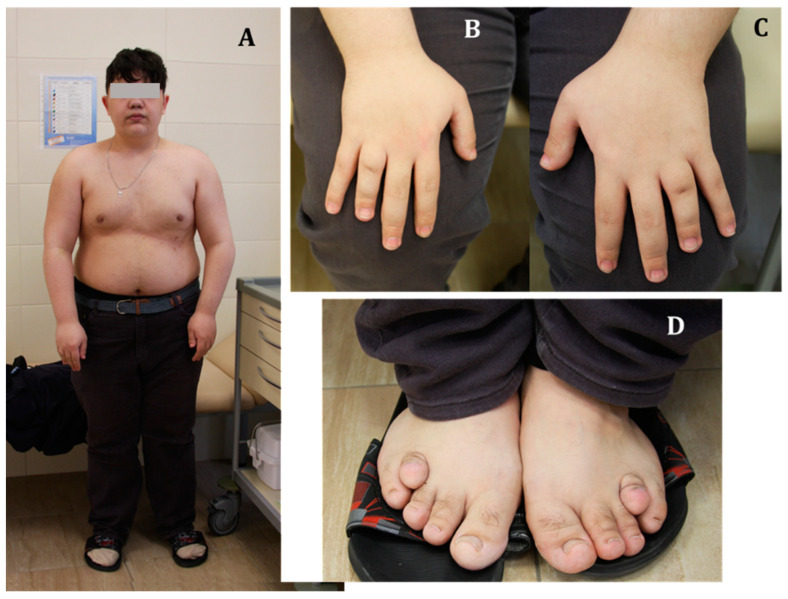
General examination of patient T. at admission. (**A**) General appearance of the patient. (**B**,**C**) The right and left hands, respectively. (**D)** Patient’s feet.

T.’s consciousness was clear; however, he did not answer all the addressed questions, and was partially oriented in space and time (he could become lost within the department). T. had stage II obesity (body weight 100 kg with height of 165 cm, BMI—36.73 kg/m^2^). Subcutaneous calcifications were not determined. Posture disturbance, shortening of the metatarsal bones of fingers 1,2,4, and 5 of both hands, and shortening and deformation of four toes were noticed (see Figure 1). Otherwise, the general examination of the patient did not reveal other significant signs. 

### 3.4. Examination Data and Treatment

Laboratory tests revealed normocalcemia (albumin-adjusted Ca was 2.16 mmol/L (2.15–2.55)), normophosphatemia (1.33 mmol/L (0.74–1.52)), normocalciuria (3.7 mmol/day (2.5–8)), and hyperphosphaturia (43.1 mmol/day (13–42)) with an increased PTH level up to 102.4 pg/mL (15–65). 25 (OH) vitamin D was above 150 ng/mL. Laboratory test results are presented in Table 2.

Due to long-term use of high doses of cholecalciferol, a mass spectrometry analysis of vitamin D metabolites in serum was performed (Table 3).

Due to laboratory results, cholecalciferol was temporarily discontinued and alfacalcidol 0.75 μg/day was initiated. After that, we confirmed the persisted normocalcemia and normophosphatemia (albumin-corrected calcium 2.13 mmol/L, phosphorus 1.3 mmol/L) with an elevated PTH level (105.8 pg/mL).

The patient was screened for PHP complications. Ultrasound revealed no signs of nephrocalcinosis/nephrolithiasis. Brain MRI demonstrated symmetrical zones of calcification in the subcortical nuclei and nuclei dentati of the cerebellum.

The patient had normal blood and urine osmolality without clinical manifestations of polyuria/polydipsia. Given the lack of evidence for the diabetes insipidus, a trial withdrawal of desmopressin was carried out, and the plasma and urine osmolality, and blood serum electrolytes, remained within the laboratory reference range. The typical MRI signal of the neurohypophysis was preserved. Thus, desmopressin was discontinued.

Considering the patient’s BMI, glucose metabolism disorders were excluded (fasting glycemia—4.69 mmol/L, HbA1c—5%). An increase in serum ACTH concentration was regarded as a consequence of multihormonal resistance. Evening salivary cortisol was elevated because of overweight-induced functional hypercortisolism. In addition, normogonadotropic hypogonadism was diagnosed (testosterone—9.59 nmol/L, LH—10.1 U/L, FSH—3.54 U/L). An andrologist scheduled a clostilbegit test on an outpatient basis.

### 3.5. Follow-Up

The patient continues dynamic follow-up at the Department. Eight months after discharge, the serum 25(OH)D decreased to 75.2 ng/mL. Nowadays, the patient takes 0.5 μg of alfacalcidol per day, with stable normocalcemia (albumin-adjusted Ca 2.15–2.2 mmol/L) and normophosphatemia (1.35–1.4 mmol/L). PTH remains increased up to 143 pg/mL. At the time of publication, a test with clostilbegit has not been performed yet.

### 3.6. Genetic Verification of Pseudohypoparathyroidism by GNAS Sequencing

**Figure 2:**
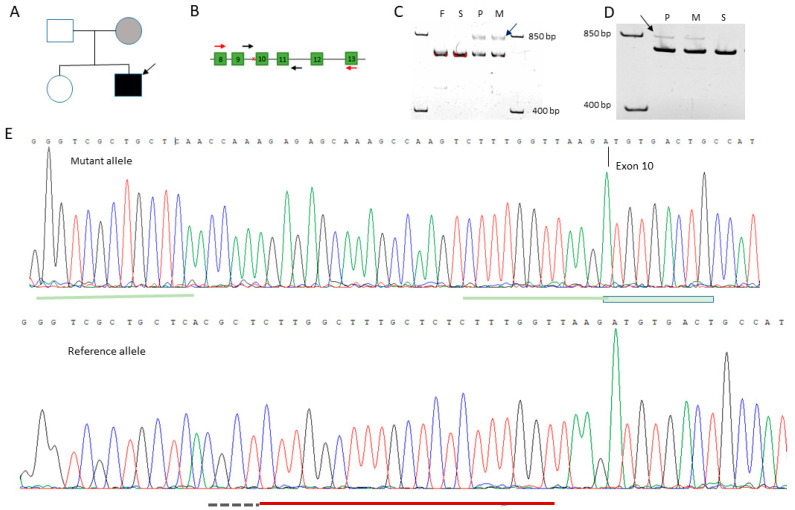
(**A**) The diagram of the investigated family. (**B**) The GNAS gene scheme (8–13 exons) with primer locations for the mutation detection (in black) and cDNA sequencing (in red). (**C**) Polyacrylamide gel image of the exon 10–11 amplicon of the GNAS gene from DNA. Arrow indicates a heteroduplex of the mutant and reference alleles. (**D**) Polyacrylamide gel image of the exon 8–13 amplicon of the GNAS gene from cDNA. Arrow indicates an additional variant found in the samples. (**E**) Sanger sequence trace of mutant and reference alleles. The green line under the mutant allele indicates overlapping regions. The red line under the reference allele denotes an inversion. The black dotted line denotes a deleted region.

In order to verify the diagnosis and provide family counseling, the patient was referred for genetic testing. Amplification of all exonic and adjacent intronic regions of the *GNAS* gene was carried out on DNA samples from the proband and his available family members, namely, his parents and sister (Figure 2A). The amplicons were subjected to Sanger sequencing, which revealed a peculiar heterozygous rearrangement in the samples of the proband and his apparently healthy mother. This variant consisted of simultaneous inversion of the 21-nucleotide sequence at the position from -5 to -25 bases from the 5’ splicing site of the 10th exon, duplication of 7 nucleotides at the 3’-end of this inverted sequence, and a deletion of 4 nucleotides at the 5’-end of it. (Figure 2E, HGVS NC_000020.11(NM_000516.7)):c.719-29_719-13delinsACCAAAGAGAGCAAAGCCAAG. This variant, initially classified by ACMG criteria as a variant of uncertain clinical significance, results in the amplicon length of the mutant allele to differ from the canonical allele by four nucleotides. In family members carrying this mutation, electrophoretic separation of exon 10–11 amplicons in the polyacrylamide gel revealed an additional band, which was attributed to the heteroduplex of the canonical and mutant alleles. (Figure 2C)

**Figure 3:**
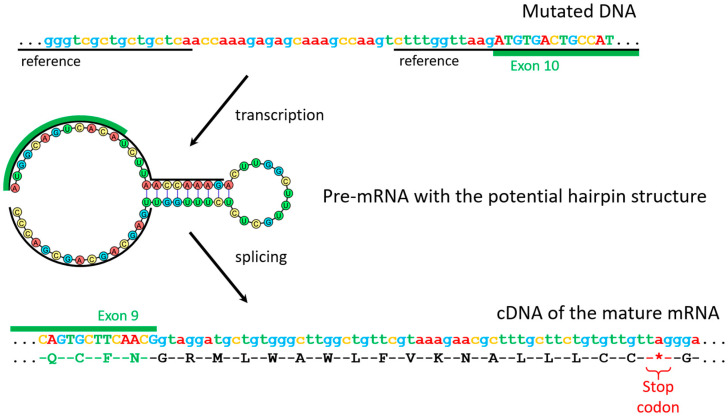
Suggested molecular mechanism of the variant’s pathogenicity.

In order to understand how the detected variant affects the gene expression, total RNA was isolated from the blood samples of the proband, his mother, and his sister. cDNA was used as the matrix for amplification of the region corresponding to 8–13 exons of the *GNAS* mRNA. Resulting amplicons were subjected to Sanger sequencing. Only the canonical mRNA sequence was found in the amplicon of the sister, while the proband’s and mother’s samples contained an amplicon with a sequence larger by about 100 nucleotides in comparison to the canonical sequence (Figure 2D). This sequencing revealed that, in addition to the exons of the gene, it includes a 108-nucleotide sequence from the mutant ninth intron that contains a stop codon. (Supplementary Figure S1) Therefore, translation of such mRNA should lead to the formation of a truncated protein (Figure 3).

## 4. Discussion

In 2016, the European EuroPHP-network proposed a new classification of the disease that takes into account all conditions associated with inactivation of the PTH/PTH1R signaling pathway (iPPSD). Three key clinical signs of PHP were chosen as the main criteria: PTH resistance, subcutaneous ossifications, and type-E brachydactyly. The minor criteria (TSH resistance, other hormonal resistances, motor and cognitive retardation or impairment, intrauterine and postnatal growth retardation, obesity/overweight, and flat nasal bridge and/or maxillary hypoplasia and/or round face) are less specific, and therefore they are considered only in combination with one or more major criteria. They also proposed numbering for both clinical features and molecular and genetic findings. Each gene was assigned a number, so patients can be ranked to a specific genetic group. This classification has already been validated in a sample of 544 patients with a genetically verified disease [12].

Despite several advantages (the distribution of disorders caused by the same mechanisms into groups, the possibility of the new data supplementation), the presented classification has some limitations. For example, it requires further confirmation—the determining of the affected allele. The suggestion that impaired PTH/PTH1R signaling should be considered a necessary condition for the development of the disease may be misinterpreted by physicians, since it excludes disturbances in cAMP-mediated signaling from other Gαs-associated receptors. Thus, there is still a need to improve the existing classifications [13].

This clinical case is an example of a PHP caused by a previously unknown GNAS mutation. According to new classification [13], our patient had iPPSD type 2. It is characterized by inactivating *GNAS* mutations and the PHP Ia phenotype.

The following features characterize PHP Ia/Ic phenotypes:

1. Multihormonal resistance, including PTH, TSH, LH, FSH, somatotropin-releasing hormone, etc. [14].
2. Phenotypic manifestations of AHO, including:
  - Roundness of the face;
  - Obesity, often ahead of other endocrine disorders [15];
  - Shortening of the IV and/or V metacarpal and metatarsal bones, and the distal phalanx of the big toe. Brachydactyly is formed in the process of accelerated closure of the growth zones and is caused by impaired signal transduction from PTH1R in chondrocytes due to a Gαs defect. In patients with GNAS inactivation, brachydactyly is not present at birth but develops over time. Shortening of the metacarpal bones leads to pits in the metacarpophalangeal joints of four of the five fingers, which is manifested by the classic sign of “knuckle, knuckle, dimple, dimple” when the hand is clenched into a fist;
  - Ectopic ossification of soft tissues. The main cause is the deficiency of Gαs in mesenchymal stem cells, which contributes to their differentiation into osteoblasts in extra-osseous areas—subcutaneous tissue and dermis, followed by the formation of new bone tissue [16];
  - Decrease in mental abilities of varying severity [17]. Impairment of cognitive functions is detected in about half of patients with PHP Ia. The cause, apparently, is a Gαs defect, but not chronic hypocalcemia, since mental abilities are preserved in patients with other forms of PHP and a low level of blood calcium.

Patient T. had both clinical and laboratory manifestations of PTH resistance. In particular, disruption of PTH/PTH1R signaling resulted in hypocalcemia and hyperphosphatemia. It is also known that disturbance of PTH/PTH1R signaling in the renal tubules leads to a decreased expression of 1α-hydroxylase (an enzyme that activates 25(OH)vitamin D), as well as sodium-dependent phosphate transporters. This leads to hypocalcemia and hyperphosphatemia in patients with PHP accompanied by elevated PTH levels in blood [18]. Hyperphosphatemia, in turn, is the cause of subcortical nuclei and nuclei dentate calcification. Endocranial calcification in the basal ganglia, as well as in the thalamus and cerebral cortex, is known as Fahr’s syndrome [19,20].

In addition, the presented case demonstrates resistance to other Gαs-associated hormones (TSH and ACTH), which is also typical for PHP Ia/ Ic types. Therefore, except the ectopic soft tissue ossification, T.’s phenotype was fully consistent with AHO. During differential diagnosis, the fact of AHO presence made the PHP1A the most plausible diagnosis in the present case, despite the absence of GNAS methylation analysis [21].

The similarity of the clinical course, laboratory results, including AHO and resistance to peptide hormones in PHP Ia and Ic creates certain difficulties in differential diagnosis. The only difference between Ia and Ic types is the degree of Gαs activity, which is reduced in IA (~50% of the normal range), but is intact in type IC [22]. For diagnostic purposes, the Gαs activity of the erythrocyte membrane is studied in vitro. In most cases, PHP Ic is considered as a type of PHP Ia. According to new iPPSD classification, all these cases are referred to as iPPSD2 [13].

Gαs mediates response to ACTH, but adrenal insufficiency is not a classic manifestation of PHPT, and only several clinical cases are described in the literature. This may be explained by biallelic expression of Gαs in the adrenal cortex [23,24,25]. In our case, there were signs of ACTH resistance (elevated ACTH level) without signs of adrenal insufficiency. This may be interpreted as subclinical ACTH resistance.

Understanding of the clinical course and the genetic origin of the disease allows us to personalize the management of patients with orphan diseases. In particular, before the diagnosis was verified, the patient received excessive doses of cholecalciferol, despite active vitamin D metabolites being a first-line treatment [26].The main goals of PHP therapy are normalization and maintenance of serum calcium. It is extremely important for avoiding hypercalcemia and, as a result, hypercalciuria, as well as for suppression of excessive PTH levels. PHP treatment requires regular follow-up of laboratory parameters and renal function. Most patients with PHP type Ia usually do not develop nephrocalcinosis [27]. However, the development of hypercalciuria is promoted, first of all, by excessive intake of calcium-increasing drugs.

In PHP, *GNAS* mutations are the most frequent cause of the disease. Some of the reported variants are located in the intronic regions near splicing sites. It was shown that these variants frequently lead to the preservation of the mutant intronic sequence in the mRNA [28,29]. In our case, we showed that the identified variant leads to the preservation of the intronic sequence containing a stop codon, which in turn leads to the truncated protein. Table 4 summarizes PHP Ia cases caused by aberrant splicing, including the present case and previously reported cases.

Thus, the presented case describes a pathogenic *GNAS* variant within an intron, outside of the donor/acceptor sites, which could be overlooked or can be difficult to interpret in conventional genetic screening tests.

The mother was shown to be an unaffected carrier of the variant, which agrees with the established inheritance of the disease. The early death of T.’s brother, who presented with a similar diagnosis but was not genetically evaluated, suggests his having been an affected carrier of the variant as well.

The effect of the mutation on the transcribed mRNA leading to the complete absence of the functional product, together with the typical inheritance pattern, proves the pathogenicity of the variant.

## 5. Conclusions

PHP patients face a wide range of conditions: from mineral metabolism changes to multihormonal resistance, ectopic ossification, and cognitive and psychomotor disorders. Management of extremely diverse clinical manifestations of both PHP and comorbid disorders creates the need for a multidisciplinary approach. Given the fact that PHP is caused by complex genetic and epigenetic defects, the method of verifying diagnosis can be long and laborious. It is important to study the presented group of diseases, and the characteristics of inheritance, including the identification of new mutations, in order to determine their pathogenicity and expected clinical manifestations. In future, this may help to develop new methods of treatment.

## Figures and Tables

**Table 1:** Dynamics of laboratory parameters of patient T. PTH—parathyroid hormone, TSH—thyroid stimulating hormone, AP—alkaline phosphatase. References were taken according to the manufacturers’ documentation.

Date	Ca total, mmol/L (2.15–2.55)	Ca ionized, mmol/L (1.03–1.29)	P, mmol/L (0.74–1.52)	PTH, pg/mL (15–65)	Na, mmol/L (136–145)	TSH, mIU/L (0.25–3.5)	AP, IU/L (40–150)	Medication
2001	-	-	-	-	-	6.7	-	Cholecalciferol 5000 IU/day Levothyroxine 12.5 mcg/day
2003	-	-	-	-	-	10.0	-	Alfacalcidol 1.2 mcg/day Levothyroxine 75 mcg/day
May 2004	1.9	-	2.4	-	-	4.3	909	Previous therapy
August 2004	1.9		2.0	-	-	-	803	Previous therapy
December 2004	1.9		1.17	-	-	-	-	Previous therapy
2005	-	-	-	-	-	7.5	-	Alfacalcidol 1.2 mcg/day Levothyroxine 100 mcg/day Desmopressin 0.2 mg twice daily
2008	1.71	0.88	2.97		127	5.29	-	Cholecalciferol 25,000 IU/day Levothyroxine 125 mcg/day Desmopressin (dose data are not available)
2010	-	-	-	-	-	15.5	-	Cholecalciferol 25,000 IU/day Levothyroxine 150 mcg/day Desmopressin (dose data are not available
2014	2.12	1.14	3.1	236.5	135.4	2.72	521	Cholecalciferol 50,000 IU/day Levothyroxine 150 mcg/day Desmopressin (dose data are not available
February 2021	-	-	-	-	-	-	-	Cholecalciferol 50,000 IU/day Levothyroxine 150 mcg/day Desmopressin 120 mcg/day

**Table 2:** Laboratory test results. ACTH—adrenocorticotropic hormone, TSH—thyroid stimulating hormone, FSH—follicle-stimulating hormone, LH—luteinizing hormone, SHBG—sex hormone-binding globulin, IGF-1—insulin-like growth factor 1, PSA—prostate-specific antigen, AP— alkaline phosphatase, LDL— low-density lipoprotein, HDL—high-density lipoprotein, ALT—alanine aminotransferase, AST—aspartate aminotransferase.

Parameter	Value	References	Parameter	Value	References
Morning ACTH	**103.9** pg/mL	7.2–63.3	Albumen	49 g/L	35–50
Evening ACTH	**26.76** pg/mL	2–25.5	Sodium	139 mmol/L	136–145
TSH	2.145 mIU/L	0.25–3.5	Chlorides	101 mmol/L	98–107
FSH	3.54 U/L	1.6–9.7	Potassium	3.9 mmol/L	3.5–5.1
LH	10.1 U/L	2.5–11	Glucose	4.69 mmol/L	3.1–6.1
Testosterone	**9.59** nmol/L	11–28.2	HbA1c	5%	4–6
SHBG	23.99 nmol/L	18.3–54.1	Creatinine	65.8 mcmol/l	63–110
IGF-1	301.7 ng/mL	119–511	Phosphorus	1.33 mmol/L	0.74–1.52
STH	0.093 ng/mL	0.02–1.23	AP	68 U/L	40–150
Blood cortisol in the morning	487.5 nmol/L	171–536	Total cholesterol	4.52 mmol/L	3.3–5.2
Evening saliva cortisol	**10.11** nmol/L	0.5–9.65	LDL	2.8 mmol/L	1.1–3
Plasma osmolality	287 mOsm/kg	280–300	HDL	1.278 mmol/L	0.9–2.6
Urine osmolality	703 mOsm/kg	300–1200	Triglycerides	1.06 mmol/L	0.1–1.7
PSA (total)	0.25 ng/mL	0–4	ALT	14 U/L	0–55
Magnesium	0.84 mmol/L	0.7–1.05	AST	17 U/L	5–34

**Table 3:** Results of vitamin D metabolites in the patient’s blood serum measurement.

Parameter	Value, ng/mL	References
24,25-(OH)2-D3	**22.6**	0.5–5.6
25-(OH)-D3/24,25-(OH)2-D3	12.654867	7–25
25-(OH)-D3	**275**	20–60
25-(OH)-D2	**0.01**	20–60
Total 25-(OH)-D	**275.01**	20–60
3-epi-25-(OH)-D3	**22.7**	1–10

**Table 4:** Examples of different GNAS splicing mutations in PHP1A.

Mutation	Location of Splicing Mutation	Effect on Pre-mRNA Splicing	Commentary	Reference
c.119_139 + 17del	Exon 1/intron 1 boundary	Partial deletion (21bp) of exon 1	The 38 bp deletion at the exon 1/intron 1 boundary comprising 21 nucleotides of the 3’-end of exon 1 and 17 nucleotides of intron 1 in the mutated allele. This eliminates the donor splice site of exon 1, giving rise to a transcript that includes intron 1. As a result, termination of translation is predicted to occur within intron 1, leading to the incorporation of at least 116 alternative amino acids into a protein product of the mutated Gsα gene.	[30]
c.313-11A > G	Intronic	Retention of intron 4	The inclusion of intron 4 in the Gαs transcript is predicted to result in premature truncation 21 codons downstream.	[31]
c.312+5G > A	Intronic	Exon 4 skipping	This change is predicted to result in aberrant splicing with removal of exon 4.	[32]
c.212 + 3_212 + 6delAAGT	Intronic	Cryptic exon inclusion	Predicted to disrupt the highly conserved 5’ splice site sequence in intron 2 and activate a cryptic splice site located 28 bp downstream of the intron.	[33]
c.719-29_719-13delinsACCAAAGAGAGCAAAGCCAAG	Intronic	Cryptic exon inclusion	Sequencing revealed that, in addition to the exons of the gene, it includes a 108-nucleotide sequence from the mutant ninth intron that contains a stop codon.	This work

## Data Availability

Available by request.

## Supplementary Materials

The following are available online at https://www.mdpi.com/article/10.3390/genes14020324/s1, Figure S1: Chromatogram from cDNA sequencing of mutant and reference alleles. Green line indicates canonical sequences of the GNAS gene 9 and 10 exons, Table S1: Primers sequences used in this work.
