# Supplementary material for: A Novel GNAS Mutation in a Patient with Ia Pseudohypoparathyroidism (iPPSD2) Phenotype
Source: Genes (Basel). 2023 Jan 26;14(2):324. doi: 10.3390/genes14020324 (PMC9956201; doi:10.3390/genes14020324)
Supplement: Supplementary file 1 [file genes-14-00324-s001.zip › genes-2146222-supplementary.pdf]

Supplementary Table S1. Primers sequences used in this work

Name	Sequence	Region of amplification
GNAS_1F	CCTCCCGGCCCGCGTGAG	DNA/ 1 exon
GNAS_1R	AAACCCCTGGCGTCGTGTAG	
GNAS_2F	GAAAGTGCTGCATCGCTACG	DNA/ 2 exon
GNAS_2R	GCAACCAGGCACGATTTTC	
GNAS_3F	TGTTCCAATTTAGCCAGAAAGG	DNA/ 3 exon
GNAS_3R	CTGCTGCAAGGTACCAAGG	
GNAS_4-5F	TTGCACAGATCCGAACCC	DNA/ 4-5 exons
GNAS_4-5R	CTGAAGTGTGGTTTGGATGC	
GNAS_6F	TCAGTAGCTTACCCCATTCCC	DNA/ 6 exon
GNAS_6R	CCAGTGGGGTAACTGGTTGG	
GNAS_7-9F	TCACTTCCGTTGAGCCTGAC	DNA/ 7-9 exons
GNAS_7-9R	CAGCGACCCTGATCCCTAAC	
GNAS_10-11F	TGGGCTTGGCTGTTCG	DNA/ 10-11 exons
GNAS_10-11R	ATATGAGAATTAGTGGGAGTGCG	
GNAS_12-13F	TCCCACCACCAAACCATAAAG	DNA/ 12-13 exons
GNAS_12-13R	GGAACATCTAAGCAAGCTGAAGG	
GNAS_r8F	CCGTGTCCTGACTTCTGGAAT	cDNA/ 8-13 exons
GNAS_r13R	AAGCTGAAGGGAAAAGGGGG	

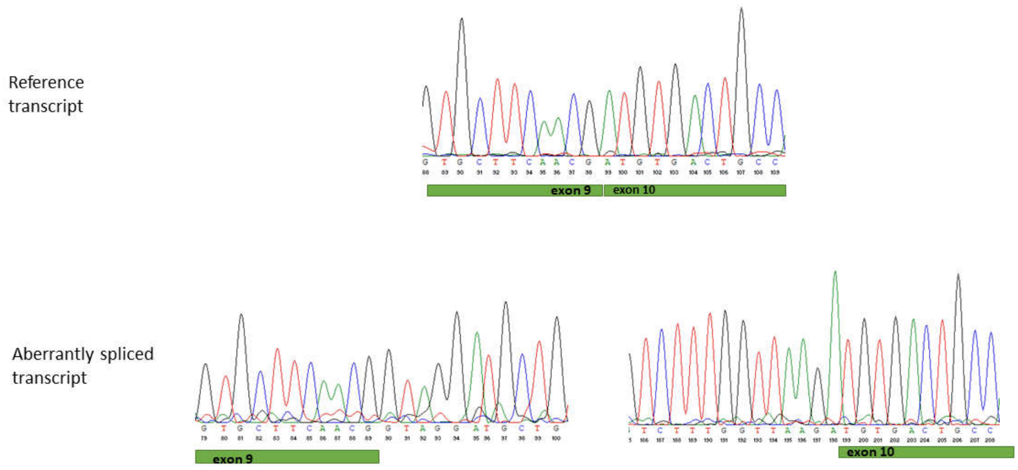

Supplementary Figure S1. Chromatogram from cDNA sequencing of mutant and reference alleles. Green line indicates canonical sequences of the GNAS gene 9 and 10 exons
